# Analysis of Schizophrenia Data Using A Nonlinear Threshold Index Logistic Model

**DOI:** 10.1371/journal.pone.0109454

**Published:** 2014-10-17

**Authors:** Zhenyu Jiang, Chengan Du, Assen Jablensky, Hua Liang, Zudi Lu, Yang Ma, Kok Lay Teo

**Affiliations:** 1 Department of Mathematics and Statistics, Curtin University, Perth, Australia; 2 Department of Statistics, George Washington University, Washington, D.C., United States of America; 3 Centre for Clinical Research in Neuropsychiatry, The University of Western Australia, Crawley, Australia; 4 Southampton Statistical Sciences Research Institute & Mathematical Sciences Academic Unit, University of Southampton, Southampton, United Kingdom; 5 Biostatistics Center, George Washington University, Washington, D.C., United States of America; University of North Carolina, United States of America

## Abstract

Genetic information, such as single nucleotide polymorphism (SNP) data, has been widely recognized as useful in prediction of disease risk. However, how to model the genetic data that is often categorical in disease class prediction is complex and challenging. In this paper, we propose a novel class of nonlinear threshold index logistic models to deal with the complex, nonlinear effects of categorical/discrete SNP covariates for Schizophrenia class prediction. A maximum likelihood methodology is suggested to estimate the unknown parameters in the models. Simulation studies demonstrate that the proposed methodology works viably well for moderate-size samples. The suggested approach is therefore applied to the analysis of the Schizophrenia classification by using a real set of SNP data from Western Australian Family Study of Schizophrenia (WAFSS). Our empirical findings provide evidence that the proposed nonlinear models well outperform the widely used linear and tree based logistic regression models in class prediction of schizophrenia risk with SNP data in terms of both Types I/II error rates and ROC curves.

## Introduction

Genetic information is useful in prediction of disease risk [1]. For example, schizophrenia is one of the most serious and frightening of all mental illnesses, and the greatest risk factor of a positive family history reflects the genetic proximity between relative and proband. It is recognized that many risk genes exist with each of small effect and each relatively common in the general population. Patients probably inherit several risk genes, which interact with each other and the environment [2] to cause schizophrenia once a critical threshold is crossed [3, page 91]. In this paper, our main objective is to propose a new class of nonlinear threshold index nonlinear logistic models, to characterize the complex links of genetic information of categorical single nucleotide polymorphism (SNP) data to the class prediction of disease risks.

The SNP data sets are high-throughput genomic data that provides useful information for identifying pathways and genes that are related to various clinical phenotypes. For example, genetic factors together with environment play a significant role in the development of schizophrenia. As reviewed by [3], while the lifetime risk in the general population is just below 1%, it is 6.5% in first degree relatives of patients [4], and it rises to more than 40% in monozygotic twins of affected people [5]. SNPs are probably the most common, and so far the best investigated genetic variations. A SNP is a DNA sequence variation occurring when a single nucleotide (A,T,C or G) differs between members of species. Each SNP can take one of the 3 forms: homozygous reference genotype; heterozygous variant genotype and homozygous variant genotype. SNPs are assumed to alter the risk for developing a particular disease. It is, however, very unlikely that any individual SNP plays an important role in the development of complex disease. Instead, multiple genes of small to moderate effect, as well as a host environmental influences are supposed to explain the differences between low and high risk groups. In practice, after recoding for analysis, the SNP data are high-dimensional and categorical.

How to efficiently utilise the genetic information of SNP data in disease classification is complicated and challenging. The complex effects of multiple genes in explaining the differences between low and high risk groups calls for a kind of nonlinear logistic regression models. General tree model [6] popular in the health sciences could be used to characterize such nonlinear interactions, but it is a kind of nonparametric method which suffers from curse of dimensionality when the dimension of the covariate vector is very high [7]. In the first author's thesis [8], it is found that the tree-based logistic model, even with a pathway-based additive form, performs worse than the linear logistic model in the class prediction of the schizophrenia risk by using the SNP data. Alternatively, extended from linear models, single index models [9], by using smoothing techniques, can be used to estimate the nonlinear factors in logistic regression when the regressor variables are continuous [10]. These semi-parametric nonlinear models are very popular in many applications. See [11] for a comprehensive survey and various applications of single-index models. To further combine the interpretability of multiple linear models and flexibility of single-index models, their hybrid, the partially linear single-index models (PLSiM), have been studied and applied for analyzing various complex data generated from biological and economic studies in the literature [12]–[15]. The first remarkable work on PLSiM can be traced back to [16], in which a backfitting algorithm was proposed to estimate parameters of interest in a more general case. [14] suggested a penalized spline estimation procedure. [13] applied the minimum average variance estimation (MAVE) [17] to PLSiM and developed an effective algorithm. More recently, [15] studied estimation in PLSiM with additional assumptions imposed on model structure. [12] proposed a profile least squares estimation procedure. But for the categorical regressors like SNP data, we can not apply these above models to capture the nonlinear interaction effects because of the categorical nature of SNPs.

In this paper, a new class of threshold index logistic regression (TILoR) models is thus proposed, which are of parametric structures combined with the dimension-reduction features as (but more general) in the semi-parametric partially linear single-index models of [10]. This method can not only use the genotype variables (SNPs) themselves to predict phenotype (complex disease) with satisfactory outcome, but also identify combinations of SNPs and quantify the importance of these interactions in SNPs. The most important advantage of the proposed model is that the model can parsimoniously reflect qualitative change of the probability when the combination of SNPs achieves a threshold, which is unknown and estimated from the data. We apply the proposed model and method for studying the SNP data set of the Western Australian Family study of Schizophrenia (WAFSS), a study dedicated to the identification of genetic interactions associated with schizophrenia. We empirically demonstrate that the proposed nonlinear models viably outperform the widely used linear or tree-based nonlinear logistic regression in class prediction of schizophrenia risk based on SNP data in terms of both Types I/II error rates, predictive accuracy and ROC curves (see Section).

The remaining of this paper is organized as follows: In Section 2, we will introduce the proposed threshold index logistic regression models. The maximum likelihood methodology to estimate the unknown parameters in the models will be suggested in Section. Section will apply the proposed model and methodology to the analysis of the schizophrenia risk classification using the SNP data from the WAFSS. In Section, the properties of the proposed methodology are then investigated with Monte carlo simulated data of moderate size. Section concludes.

## The Models

Logistic regression is extensively popular with dichotomous responses in numerous disciplines [18]. In particular, biostatistical methods are grounded in the analysis of binary and count data and the logit plays a central role in the analysis of the binary data in such as case-control study to assess relative risks of disease [19]. Under linear logistic regression structure, various methods and applications, in the literature, have been well developed no matter if the predictor variables are discrete or continuous; see, for example, [18] and [20] for comprehensive reviews and also [21] for the recent application in biostatistics. However, beyond the linear structure, a logistic regression becomes far more difficult and complex to apply when the genetic information of categorical data is considered.

In this paper, we propose a model of logistic regression allowing for a nonlinear structure for categorical genetic information. Suppose 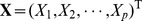 consists of a large number of gene SNPs, say 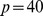 SNPs as our regressors in our real data example of Section, which are used to predict the phenotype 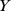 that takes on binary values in a case-control study. Consider the model: 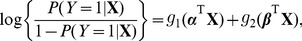
(1)where 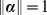, 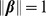, 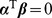, and the first non-zero components of 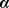 and 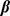 are positive, for model identifiability, and 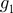 and 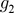 are two one-dimensional nonlinear functions which are modelled by two stepwise linear functions through threshold effects as follows: 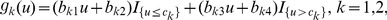
(2)where *b_ki_*'s and *c_k_*'s are unknown parameters to be estimated. Here we have extended the idea of threshold (auto)regression of [22], [23] in nonlinear time series analysis to the nonlinear genomic analysis of SNP data which are categorical. Thus, (1) and (2) form an additive threshold index logistic regression (A-TILoR) model 
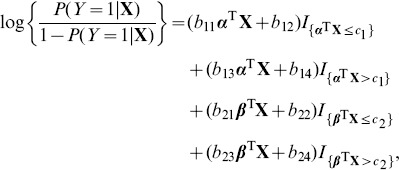
(3)with 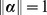, 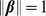, 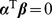, and the first non-zero components of 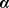 and 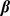 being positive.

The motivation of proposing the above models lies in twofold. Firstly, Model (3) is intuitively appealing. Notice that many risk genes exist with each of small effect [3], which interact with each other to cause schizophrenia once a critical threshold is crossed. It appears that the indices of 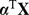 and 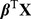 in these models could just reflect the interactive effects of individual risk genes, which are combined together forming regimes in the form of these indices, while the thresholds in (2) would indicate the threshold effects of the regimes. Secondly, as referees commented, why do we use two functions 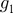 and 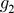, not one or three functions, in model (1)? This is because model (1) with two functions 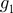 and 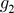 does take the model with one function as a special case (say 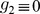) and is significantly more parsimonious than the model with three functions, in view of the large dimension 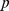 of 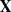 in applications (say 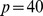 in Section 4). We shall show in Section 4 that model (3) viably outperforms the linear logistic regression and random forest in the analysis of the SNP data in the class prediction of the schizophrenia risk.

## Maximum Likelihood Estimation

Let 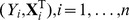, be random vectors that are independently and identically distributed as 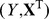.

### Subsection 1 Model parameters estimation

First of all, we look at the MLE for the A-TILoR model (3). Write 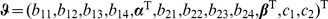 and 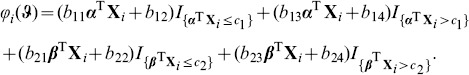
(4)


The log-likelihood can be expressed as: 
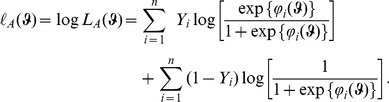
(5)


Maximizing the log-likelihood (5) with respect to 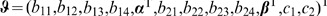 subject to the constraints 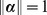, 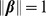 and 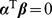 leads to the MLE 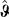 of 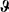. For convenience of calculation, in general we can apply the method of Lagrange multipliers to turn the maximization of (5) with the constraints into a maximisation of the following function 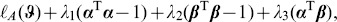
(6)with respect to 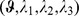.

Note that the log-likelihood (6) is not differentiable with respect to 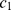 and 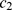 as well as 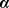 and 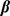 owing to (4). Therefore the widely used iteration procedure in optimization such as Newton-Raphson algorithm cannot be used here. We apply the downhill simplex method for the maximization of the log-likelihood (6), which does not require the multi-dimensional objective function of the optimization to be differentiable; for details, the reader is referred to [24, pp413] on the method and code.

In our numerical experiments, we used the R version of the standard downhill simplex method, translated from the C code of [24]. According to our experience, this algorithm works rather stably and fast in convergence with well specified initial values of the vector 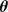 or 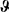, for which we need experimental tries to achieve a global maximum as done in using other optimization algorithms. In our numerical examples below, our experimental tries were based on many different initial values generated randomly, with which we can identify possible global maximum by refining the initial values in the downhill simplex algorithm.

### Subsection 2 Bootstrap estimation of the standard deviation of parameter estimates

We now evaluate whether the estimated value of an unknown parameter is significantly away from zero or not, i.e., testing whether we can reject the null hypothesis that the estimated parameter is equal to zero. This requires the knowledge of the standard deviation of the estimator of each parameter.

One way to estimate the standard deviation of the estimator of each unknown parameter is through estimating the asymptotic variance of the estimator of the parameter, which can be established by following the argument of [25]. However, asymptotic variance is based on the assumption that the sample size tends to infinity, which may be difficult to apply sometimes. We therefore suggest to estimate the standard deviation by using the bootstrap.

Given the observations 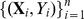, we denote the MLE of unknown parameters by 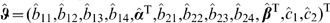 Then, the bootstrap procedure works as follows:

1. Generate a bootstrap sample of size 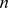:
  - For the *i*-th observation 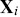, calculate 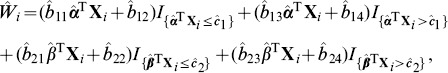
and 
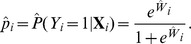

  - Generate the *i*-th bootstrap observation 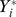 from a binomial distribution 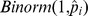.
  - For 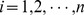 in Steps a) and b), a bootstrap sample of size 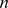, 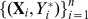, is generated.
2. Obtain a bootstrap MLE of 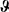 using the bootstrap sample of size 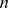, 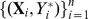:

The estimation is calculated by using the method provided in Section 3.1, where we use 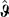 as the initial values of the parameters in the maximum likelihood procedure for the bootstrap sample 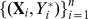. Denote the unknown parameters of the bootstrap MLE by 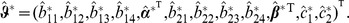

1. Repeat Steps 1) and 2) 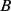 times. Denote the 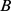 bootstrap estimates of 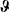 by 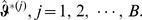

2. The standard deviation of the k-th component of 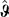 is calculated as 
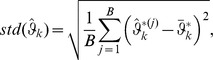
where 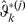 is the *k*-th component of 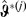 obtained in Step 3), and 
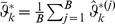
.

The main burden of computation in the above bootstrap procedure lies in Step 2). Here the maximisation of the likelihood for each bootstrap sample by using the downhill simplex method, given at the end of Section 3.1, needs well specified initial values of the vector 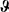, which may require a bit time-consuming experimental tries in general if we have no information on the actual value of the vector 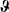. Luckily, in the bootstrap, a simple way to reduce this computation burden is to fully utilise the estimator 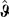 because the bootstrap sample is generated based on this data-based estimator, and therefore we can well specify the initial values of the vector 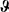 in Step 2) by adding small randomly-generated (vector) values to 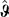.

## Prediction of Schizophrenia Risk Using SNPs Data

We now apply the proposed methodology for analysis of a real SNP data set in the schizophrenia study conducted in Western Australia, which is dedicated to identification of the genetic interactions associated with schizophrenia.

The data set is from the Western Australian Family Study of Schizophrenia (WAFSS) case-control study that started from 1996 and is still continuing today. The WAFSS study population includes 496 Western Australians of European descent, in which there are 325 members affected by schizophrenia (cases), and 171 population controls. Genotyping was conducted on 23 selected genes according to neurological knowledge and research interests. A total of 1022 SNPs was found. We first applied the OR (odds ratio) principle [1, pp70] to choose important SNPs, from which 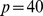 SNPs are selected at the significance level (i.e, Type I error rate) of 5%. We use these 40 SNPs as our regressors, denoted by 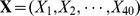; see Table 1 for these 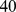 SNPs.

**Table 1:** WAFSS Study: Estimated coefficients 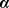, 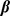 and their standard deviations (s.d.).

SNP	*α* (s.d.)	*β* (s.d.)
*X*_1_(rs8074995)	0.0058 (0.0042)	0.1393 (0.0050)
*X*_2_(rs439401)	0.3166 (0.0052)	0.1727 (0.0051)
*X*_3_(rs10774517)	−0.0797 (0.0041)	−0.1082 (0.0044)
*X*_4_(rs7960673)	−0.0161 (0.0043)	−0.0541 (0.0044)
*X*_5_(rs6490272)	0.0004 (0.0048)	0.1058 (0.0042)
*X*_6_(rs534455)	0.1194 (0.0042)	0.1804 (0.0047)
*X*_7_(rs486706)	−0.0343 (0.0055)	0.0503 (0.0047)
*X*_8_(rs694060)	−0.0905 (0.0047)	0.0630 (0.0042)
*X*_9_(rs12128305)	−0.1112 (0.0042)	0.0810 (0.0048)
*X*_10_(rs11207007)	0.1359 (0.0036)	−0.0288 (0.0054)
*X*_11_(rs6687842)	−0.0203 (0.0040)	−0.0993 (0.0050)
*X*_12_(rs10047071)	−0.0531 (0.0051)	−0.2190 (0.0054)
*X*_13_(rs17424216)	−0.2258 (0.0059)	0.0227 (0.0040)
*X*_14_(rs2991515)	−0.0350 (0.0047)	0.0800 (0.0048)
*X*_15_(rs11581152)	0.1220 (0.0051)	0.0241 (0.0042)
*X*_16_(rs852787)	−0.1378 (0.0060)	0.0976 (0.0039)
*X*_17_(rs9432024)	−0.2081 (0.0056)	0.1916 (0.0043)
*X*_18_(rs11122357)	0.0368 (0.0056)	−0.3270 (0.0042)
*X*_19_(rs877984)	0.1109 (0.0046)	0.0651 (0.0046)
*X*_20_(rs1400316)	−0.0826 (0.0050)	−0.2375 (0.0049)
*X*_21_(rs1399622)	−0.0473 (0.0036)	−0.0544 (0.0052)
*X*_22_(rs17507049)	−0.2784 (0.0050)	0.1464 (0.0046)
*X*_23_(rs7121214)	0.1016 (0.0052)	−0.0622 (0.0049)
*X*_24_(rs7928038)	0.1064 (0.0045)	−0.3077 (0.0042)
*X*_25_(rs10501563)	−0.1824 (0.0050)	−0.1235 (0.0048)
*X*_26_(rs1940078)	−0.0405 (0.0060)	−0.4768 (0.0041)
*X*_27_(rs1943699)	0.2444 (0.0049)	−0.0024 (0.0051)
*X*_28_(rs6592211)	−0.1094 (0.0048)	−0.1192 (0.0035)
*X*_29_(rs17203281)	−0.5100 (0.0053)	−0.0415 (0.0050)
*X*_30_(rs1615640)	−0.1139 (0.0047)	0.0162 (0.0047)
*X*_31_(rs11220082)	−0.0795 (0.0050)	−0.1194 (0.0047)
*X*_32_(rs931671)	−0.2502 (0.0048)	0.1427 (0.0048)
*X*_33_(rs17281921)	0.0332 (0.0043)	−0.0568 (0.0039)
*X*_34_(rs1978198)	0.0342 (0.0055)	0.2519 (0.0048)
*X*_35_(rs2711881)	−0.0555 (0.0048)	−0.1884 (0.0045)
*X*_36_(rs2528865)	0.0770 (0.0051)	−0.0204 (0.0051)
*X*_37_(rs10248053)	−0.1033 (0.0056)	0.1180 (0.0058)
*X*_38_(rs2283029)	0.0845 (0.0049)	0.2366 (0.0050)
*X*_39_(rs1454626)	−0.3238 (0.0045)	0.0979 (0.0043)
*X*_40_(rs1022307)	0.0410 (0.0043)	−0.0302 (0.0047)

### Subsection 3 Analysis based on the A-TILoR model

We apply the A-TILoR model to analysis of the WAFSS schizophrenia SNP dataset, with 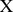 of dimension 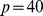: 
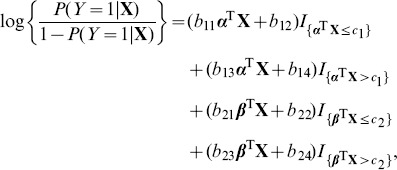
(7)where 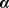 and 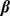 are of the identifiability conditions in model (3). Then, we estimate the unknown parameters by maximum likelihood method and the standard deviation of the estimator of each parameter by using a bootstrap procedure, as introduced in Section 3. The estimated values of the coefficients 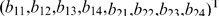, 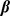 and 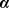 in model (7) and their bootstrap based standard deviations (s.d.), with the bootstrap sample size equal to 100, are reported in Table 2, and the estimated coefficient 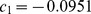 (s.d.: 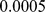) and 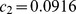 (s.d.: 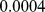).

**Table 2:** Estimated coefficients 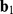, 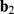 and their standard deviations calculated by bootstrap method in TILoR model for the WAFSS schizophrenia data set.

**b**_1_ = (*b*_11_,*b*_12_,*b*_13_,*b*_14_)	0.0274	0.4358	−2.7377	1.3744
s.d. (bootstrap)	0.0139	0.0873	0.0561	0.0547
**b**_2_ = (*b*_21_,*b*_22_,*b*_23_,*b*_24_)	0.0260	−0.0748	2.4239	0.4685
s.d. (bootstrap)	0.0281	0.0875	0.0629	0.0553

In genetic analysis, the individual SNPs make contributions through interactions. Our indices in the TILoR model confirm that the individual SNPs' contributions are made through such regime indices 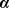 and 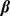 (Table 1). All the components of the index vectors 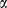 and 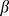, except the coefficients of 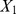 (SNP rs8074995) in 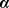 and that of 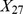 (SNP rs1943699) in 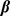, are significantly different from zero at the significance level (that is, the allowed Type I testing error rate) of both 5% and even 1%, or equivalently at the confidence level of both 95% and 99%, respectively. Schizophrenia is a complex disorder. There are multiple susceptibility genes, each with small to modest effects that interact with each other and environmental factors to influence susceptibility for this disease. It is accepted that for each gene, more than one SNP shows association with schizophrenia, but rarely are data from individual SNPs highly significant [26]. Table 1 provides an explicit quantitative proof to this biological understanding of schizophrenia using the proposed threshold index logistic regression model. For reference, in Table 3, we have also provided the larger components of 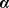 and 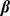 whose absolute values are greater than 0.2 and their corresponding gene: SNP names. It looks that these genes: SNPs may play a larger part in deciding the threshold effects.

**Table 3:** WAFSS Study: The components of 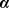 and 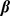 whose absolute values are greater than 0.2.

Component of X	(Gene:SNP)	Component of *α*
*X*_2_	(APOE:rs439401)	0.3166
*X*_13_	(DAB:rs17424216)	−0.2258
*X*_17_	(DISC1:rs9432024)	−0.2081
*X*_22_	(DLG2:rs17507049)	−0.2785
*X*_27_	(DLG2:rs1943699)	0.2444
*X*_29_	(DLG4:rs17203281)	−0.5099
*X*_32_	(NUDEL:rs931671)	−0.2502
*X*_39_	(VLDLR:rs1454626)	−0.3238

Component of X	(Gene:SNP)	Component of *β*
*X*_12_	(DAB:rs10047071)	−0.2190
*X*_18_	(DISC1:rs11122357)	−0.3270
*X*_20_	(DLG2:rs1400316)	−0.2375
*X*_24_	(DLG2:rs7928038)	−0.3077
*X*_26_	(DLG2:rs1940078)	−0.4768
*X*_34_	(RELN:rs1978198)	0.2519
*X*_38_	(RELN:rs2283029)	0.2366

Regarding the thresholds, the values 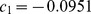 and 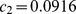 appear near 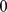, but they are still very significant, as the confidence intervals, i.e., the values of 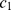 and 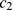 plus their three times standard deviations calculated by bootstrap method, respectively, still do not include 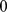.

We can also calculate the values of the indices of 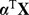
's and 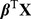
's, respectively. Compared with the thresholds 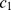 and 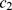, it follows that under the *α*-regime, there is a high empirical probability (90.32%) that the values of 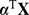 are less than the threshold 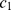, while under the *β*-regime, the empirical probability of 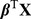 less than the threshold 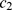 is 66.33%.

**Figure 1:**
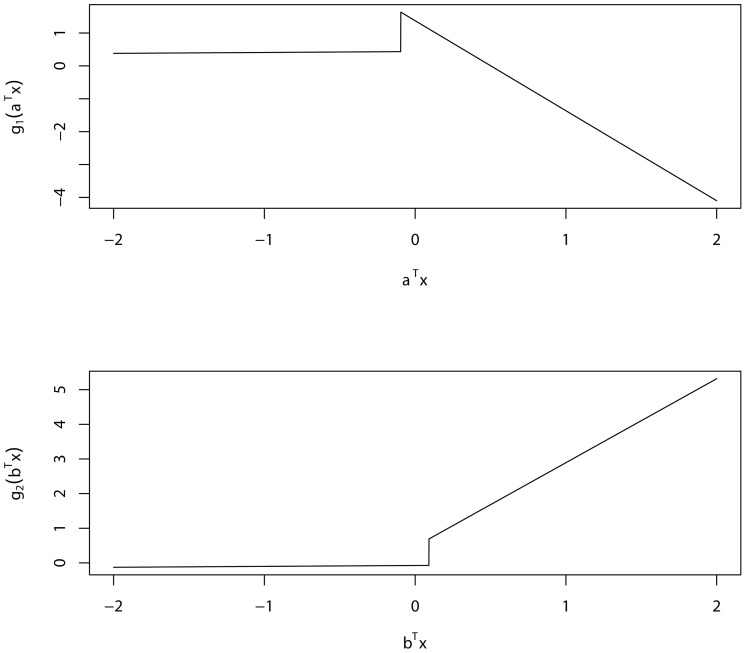
TILoR model for general schizophrenia: The plot of the functions *g_1_* and *g_2_*, respectively.

By looking at the functions 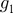 and 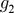 in (2), which are plotted in Figure 1, it is apparent that when the regime indices are lower than the corresponding thresholds, the impacts of the regimes are stable, but when indices are greater than the thresholds, the impacts become viably significant. This is consistent with the biological fact that the risk genes interact with each other to cause schizophrenia once a critical threshold is crossed [3]. If combining this with the fact stated above that the majorities of the index variables are less than the two thresholds (90.32% for the *α*-regime and 66.33% for the *β*-regime), it follows that the impacts in most of cases of the index variables are small; only if the regime indices are greater than the corresponding thresholds will they have significant impact, but that probability is relatively lower, with the probability of 9.68% in the *α*-regime and 33.67% in the *β*-regime. Figure 1 also provides a visual exhibition of the nonlinear feature of the impact on schizophrenia of SNP data sets. It appears that the *β*-regime plays more important role than the *α*-regime in causing schizophrenia.

### Subsection 4 Comparison with other models by Cross-Validation

In this subsection, using cross-validation, we further demonstrate the performance of our proposed A-TILoR model in comparison with some popular logistic regression models, including generalized linear model and the random forest method.

We first examine the performance of our A-TILoR model in comparison with generalised linear model in R (GLM is referred to the linear logistic regression below). We will show that our proposed TILoR method (simply denoted as TILoR below) performs viably better than the GLM and random forest.

We have carried out the comparison through cross-validation testing. It is known that the resubstitution estimate of predictive accuracy, derived by direct application of model predictions to the data from which the regression relationship is derived, gives, in general, an optimistic assessment. Because there is a mutual dependence between the model prediction and the data used to derive that prediction, an ideal is to assess the performance of the model on a new data set. The data that are used to develop the model from the training set, while the data on which predictions are tested form the test set. Cross-validation extends the training/test set approach. The data are divided into 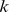 sets (or folds), where 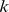 is typically in the range of 3 to 10. Each of the 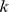 sets becomes in turn the test set, with the remaining data forming the training set. The predictive accuracy assessments from the 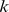 folds are combined to give a measure of the predictive performance of the model. This may be done for several different measures of predictive performance. Here we use a 3-fold validation with special considerations based on the case-control character. For the general schizophrenia data set (325 cases and 171 controls), we use a random number sampling system to divide the case data into three equal groups, and control data into three equal groups. Then we combine the case groups and the control group to form three folds. For each of the three folds, it is set aside as the test data, with the remaining data making up the training data. In each time, there are 108 cases and 57 controls in the test set, and 217 cases and 114 controls in the training set.

According to the experts from the WAFSS, the source of the data in this analysis, it is generally accepted that schizophrenia's broad heritability is about 80% (c.f., [27]). Therefore, 80% is naturally the approximate upper limit of accuracy of models using genotypes only. In other words, without using other information such as phenotypes, whatever modelling technique applies, the accuracy rate is not supposed to be higher than 80%. If we consider 50% as a model-worthy lower limit accuracy, the interval (50%–80%) gives an idea what the accuracy rate will be in. That gives us an idea about what to expect.

In Table 4, we report the comparison between the GLM and the TILoR from the predictive accuracy and the Type I and Type II error rates for the schizophrenia.

**Table 4:** WAFSS Study: Type I, Type II errors rates, predictive accuracy rates, and area under the curve (AUC) based on cross-validation estimate using GLM models, TILoR models, and random forest (RF) method.

		Fold1	Fold2	Fold3	Average
TILoR	Type I error	38.59%	36.84%	21.05%	32.16%
	Type II error	25.92%	31.48%	28.70%	28.70%
	predictive accuracy	69.69%	66.67%	73.94%	70.10%
	AUC	0.812	0.812	0.791	0.805
GLM	Type I error	52.63%	57.89%	70.17%	60.23%
	Type II error	23.14%	20.37%	15.74%	19.75%
	predictive accuracy	66.67%	66.67%	65.45%	66.26%
	AUC	0.774	0.774	0.774	0.774
RF	Type I error	63.16%	77.19%	77.19%	72.51%
	Type II error	8.33%	5.56%	3.70%	5.86%
	Prediction accuracy	72.73%	69.70%	70.91%	71.11%
	AUC	0.688	0.702	0.732	0.707

**Figure 2:**
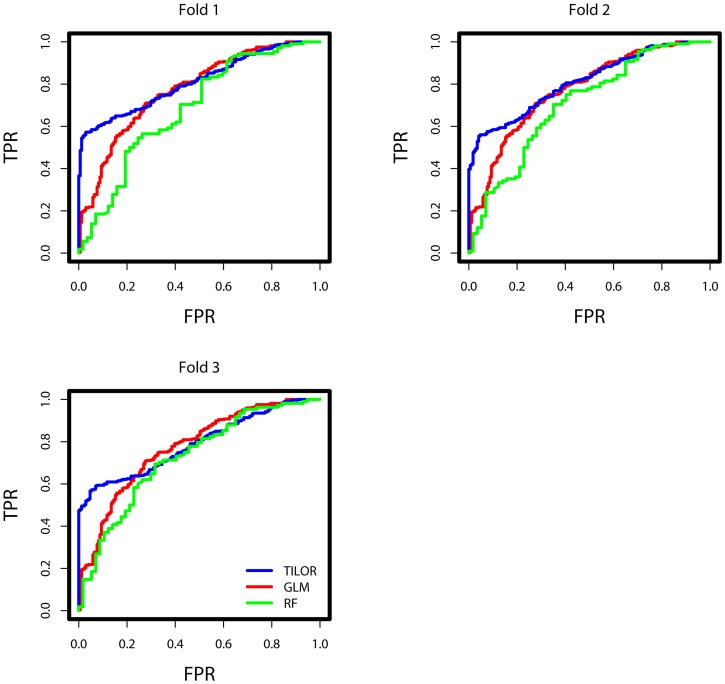
The ROC curves based on three methods/models (TILoR: Blue line; GLM: Red line; random forest: Green line) corresponding to folds 1–3.

From the above tables, we may summarize that: From the predictive accuracy perspective, the TILoR obviously performs better than the GLM in Table 4, also close to the up-limit of 80% for schizophrenia prediction (genotype only). From the perspective of the Type I and Type II error rates, the problem with the GLM is that it has a too ideal type II error but far too worse type I error (60.23% cross-validation error) in Table 4. The bad performance on type I error has made GLM itself unsuitable to be used as a practical model for schizophrenia. In contrast, in the same tables, using TILoR, both the type I error (32.16%) and type II error (28.70%) are stable and close to the 20% lower limit of the error rate. Therefore, TILoR is an eligible and nice predictor for schizophrenia classification. We have also depicted the receiver of characteristic (ROC) curves based on TILoR (solid line), GLM (dotted line), and random forest (RF; dashed line) in Figure 2, and corresponding area under curve (AUC) values in Table 4. These curves and AUC values indicate that TILoR model is uniformly superior to the counterparts. Specifically, the AUC values based on TILoR, GLM, and RF equal to 0.805, 0.774, and 0.707, respectively. In short, our TILoR viably outperforms the popular GLM method in class prediction of schizophrenia risk using SNPs data.

## A Monte Carlo Simulation Study

In this section, we are first examining the finite sample performance of the proposed estimators of maximum likelihood method for the unknown parameters in the A-TILOR model (3) by Monte Carlo simulations.

In real application of genomic data analysis, the dimension 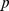 of the predictor vector is quite large, and the predictor variables are categorical with SNP data. To accommodate these scenarios, we consider the A-TILOR model, used for simulation, of the form (3) with 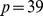, and 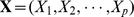, with 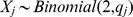, and 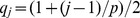, for 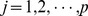, where we assume that 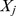
's are linearly independent with each other. We take the parameters in the model detailed below: 
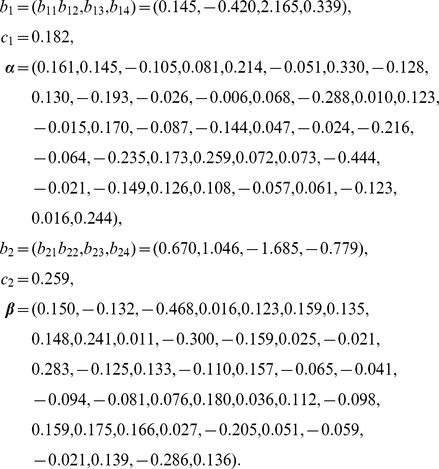



We first simulate an independent sample of size 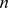 of random vector 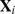 with its *j*th component 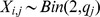, for 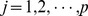, and 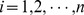. Then, for each 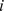, we calculate 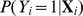 according to (3), and thus, we simulate 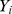 from the Bernoulli trial with probability equal to 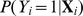.

**Figure 3:**
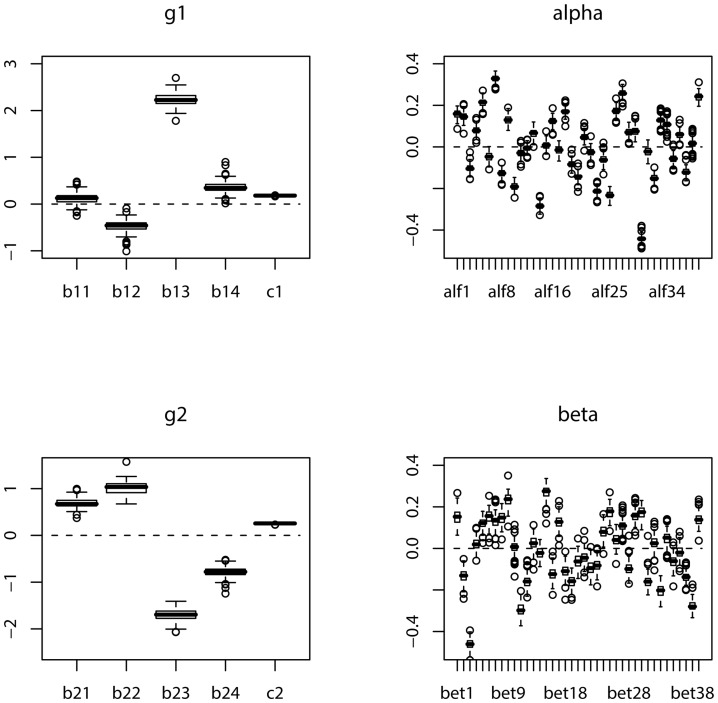
Boxplot of the estimates of the parameters in *g_1_*, 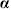 **,** *g_2_* and 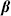 based on 100 simulations: 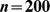.

**Figure 4:**
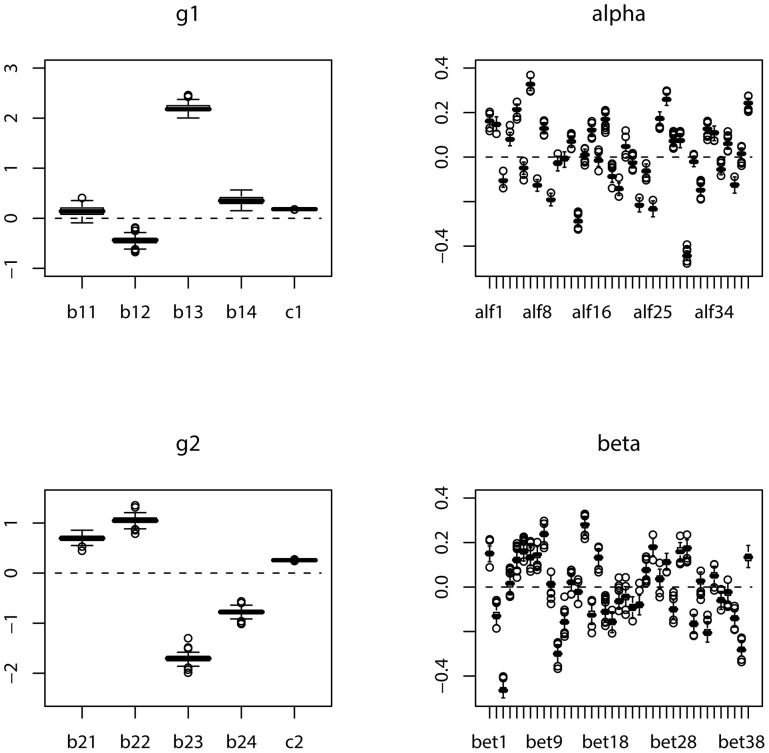
Boxplot of the estimates of the parameters in *g_1_*, 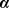, *g_2_* and 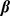 based on 100 simulations: 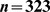.

**Figure 5:**
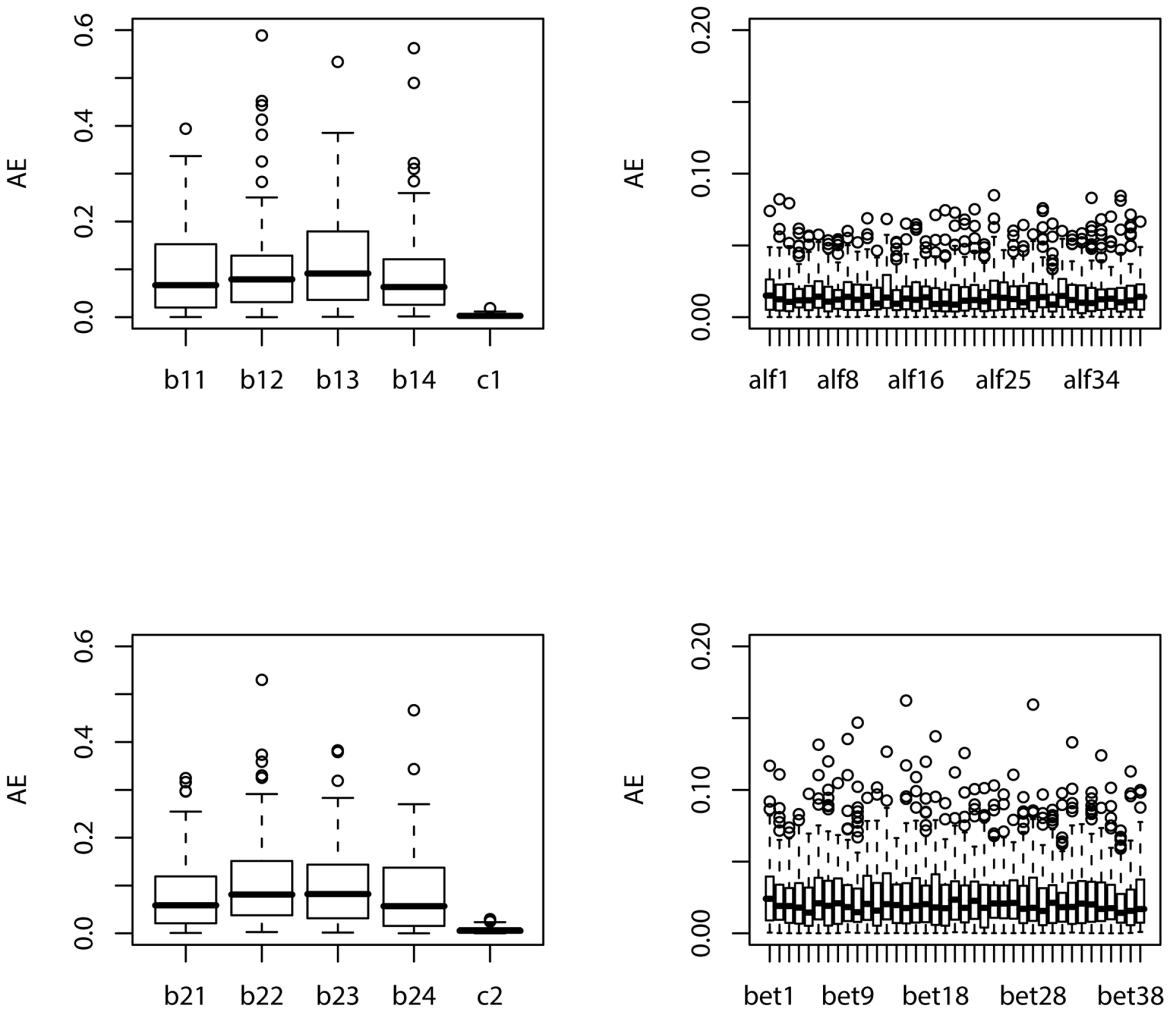
Boxplot of the absolute errors (AEs) of the estimates of the parameters in *g_1_*, 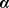, *g_2_* and 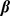 based on 100 simulations: 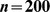.

**Figure 6:**
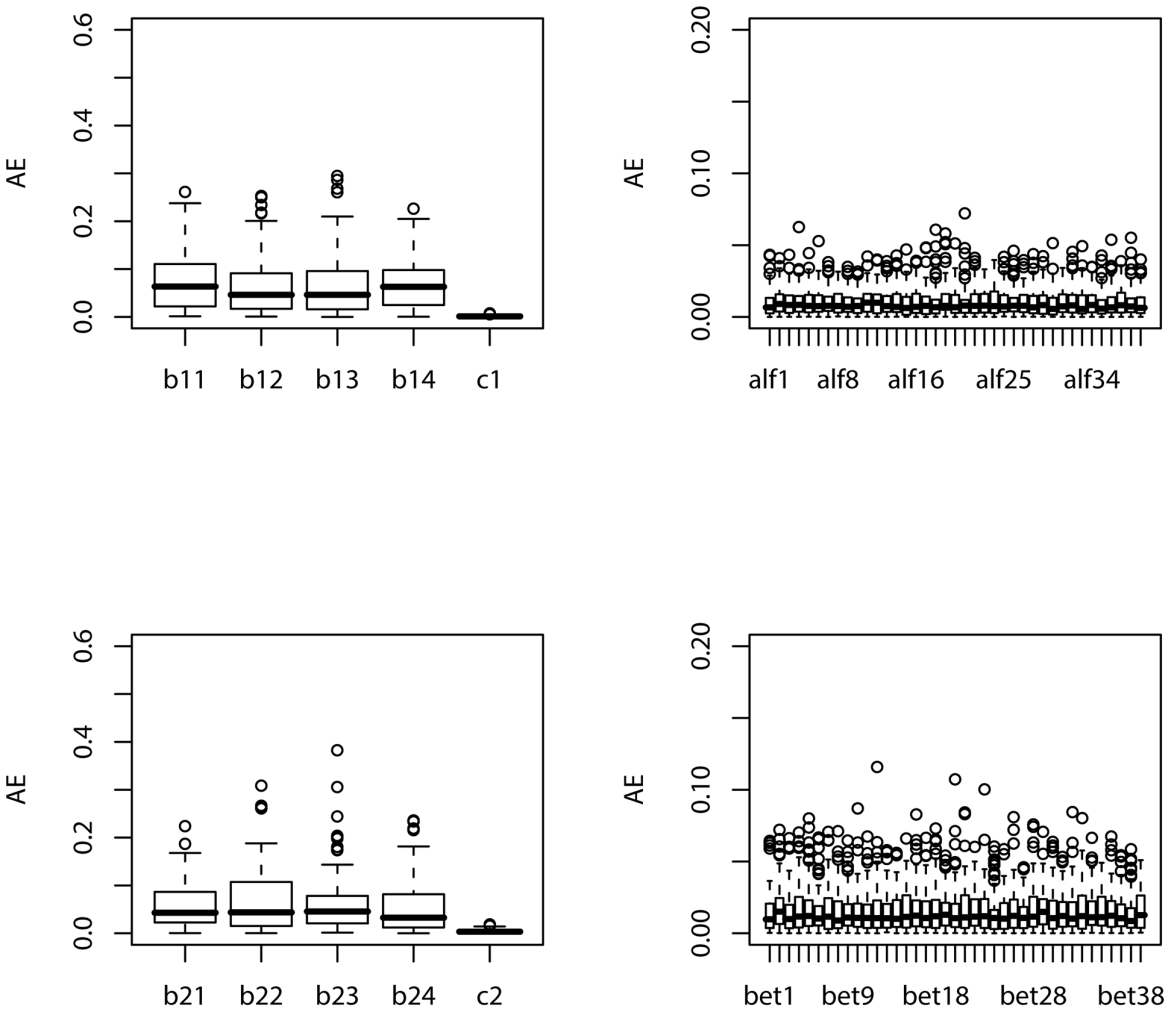
Boxplot of the absolute errors (AEs) of the estimates of the parameters in *g_1_*, 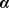, *g_2_* and 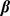 based on 100 simulations: 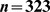.

For each simulated sample, we apply the suggested maximum likelihood method to estimate the parameters. We repeat the simulation 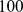 times for each of the two cases of sample size 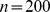 and 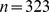, respectively. The boxplots of the estimates of the parameters in *g_**1**_*, 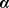, *g_**2**_* and 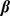 based on 100 simulations are displayed in Figures 3 and 4, for the cases of sample size 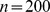 and 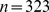, respectively. In order to assess the precision of the estimate for each of the parameters, the absolute errors of the estimates of the parameters based on 100 simulations are also depicted in boxplot in Figures 5 and 6 for the cases of sample size corresponding to those in Figures 3 and 4, respectively.

From these figures, we can conclude that as the sample size increases, the absolute error of the estimate significantly decreases. Comparing Figure 4 with Figure 3, the boxplot becomes much narrower for each parameter in Figure 4 than that in Figure 3. This also clearly follows by comparing Figure 6 with Figure 5. It looks apparent that the suggested methodology for the samples of size 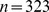 used in Figure 4 and Figure 6 is quite satisfactory for the proposed model even with a large predictor vector of dimension 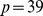. This sample size is close to that of the training data set used in cross-validation in Section 4.2.

## Conclusion and Discussions

A common and important task in genetic association studies is the identification of SNPs and SNP interactions associated with an interest, for example, a disease. Because SNP interactions are assumed to be more influential than individual SNPs, there is a need for a method to capture such complex nonlinear interactions. In this paper, we have extended the idea of threshold (auto)regression of [22], [23] in nonlinear time series analysis to the nonlinear genomic analysis of SNP data which are categorical, and we have proposed a new class of threshold index logistic regression(TILoR) models, including partially linear and additive TILoR models, to quantify the SNPs and SNP interaction for classification in case-control studies. We have provided a maximum likelihood methodology to estimate the unknown parameters, which is shown, via Monte carlo simulation, to be applicable with moderate-size samples.

Empirical study by applying the TILoR model to the schizophrenia SNP data has found that our TILoR model outperforms linear logistic model and random forests in terms of the Type I/II errors, cross-validation predictive accuracy rates, area under curve. The accuracy for schizophrenia prediction based on the TILoR model, random forest, and GLM are 70.10%, 71.11%, and 66.26%. They are similar with the first two slightly better. However, the Type I errors based on random forest and GLM are substantially larger than the Type I error based on the TILoR model although their Type II errors are smaller. Note that the Type I errors for both random forest and GLM are greater than 50%. Furthermore, the AUC based on the TILoR is higher than the AUC based the GLM and random forest. Therefore the result of the cross-validation prediction for schizophrenia with our proposed TILoR model is very encouraging.

Our TILoR schizophrenia prediction has the potential to becoming a part of medical diagnostic and disease risk management process. The medical diagnosis in psychiatry is problematic. Apart from the fact that there are differing theoretical views toward mental conditions, there are few lab tests available. Our prediction is based on the SNP genotype data alone, meaning that only a drop of blood taken from a participant will be sufficient for genotyping. The final TILoR model involves about 40 SNPs on 12 genes, which dramatically reduces the cost of genotype and therefore, the cost of the prediction. In particular, for children coming from a schizophrenia family, our findings could provide a disease risk reference to their life style chosen. For example, late adolescence and early adulthood are peak periods for the onset of schizophrenia. At this stage, avoiding environmental disadvantageous influences will be a sensible and rational way to better manage disease risk.

## Supporting Information

Dataset S1 (TXT)
